# Integrative Structural Biomechanical Concepts of Ankylosing Spondylitis

**DOI:** 10.1155/2011/205904

**Published:** 2011-12-18

**Authors:** Alfonse T. Masi, Kalyani Nair, Brian J. Andonian, Kristina M. Prus, Joseph Kelly, Jose R. Sanchez, Jacqueline Henderson

**Affiliations:** ^1^Department of Medicine, University of Illinois College of Medicine, Peoria, IL 61656, USA; ^2^Department of Mechanical Engineering, Bradley University, Peoria, IL 61625, USA; ^3^Department of Physical Therapy & Health Science, Bradley University, Peoria, IL 61625, USA; ^4^Department of Electrical and Computer Engineering, Bradley University, Peoria, IL 61625, USA

## Abstract

Ankylosing spondylitis (AS) is not fully explained by inflammatory processes. Clinical, epidemiological, genetic, and course of disease features indicate additional host-related risk processes and predispositions. Collectively, the pattern of predisposition to onset in adolescent and young adult ages, male preponderance, and widely varied severity of AS is unique among rheumatic diseases. However, this pattern could reflect biomechanical and structural differences between the sexes, naturally occurring musculoskeletal changes over life cycles, and a population polymorphism. During juvenile development, the body is more flexible and weaker than during adolescent maturation and young adulthood, when strengthening and stiffening considerably increase. During middle and later ages, the musculoskeletal system again weakens. The novel concept of an innate axial myofascial hypertonicity reflects basic mechanobiological principles in human function, tissue reactivity, and pathology. However, these processes have been little studied and require critical testing. The proposed physical mechanisms likely interact with recognized immunobiological pathways. The structural biomechanical processes and tissue reactions might possibly precede initiation of other AS-related pathways. Research in the combined structural mechanobiology and immunobiology processes promises to improve understanding of the initiation and perpetuation of AS than prevailing concepts. The combined processes might better explain characteristic enthesopathic and inflammatory processes in AS.

## 1. Introduction

Since 1950, ankylosing spondylitis (AS) had been reported to occur far more prevalently (circa 30 times) among relatives of spondylitis patients than among control subjects [1]. A hereditary risk factor was implicated by such familial findings [1], which have been supported by subsequent studies [2–4]. In 1973, a strong genetic predisposition was confirmed by the discovery of the remarkably high association of AS with HLA-B27 [2, 3].

Beside the recently reviewed expanded genetic risks [4], the multifactorial causation of AS was indicated early by a definite male preponderance of clinical disease [5–8]. The male preponderance was particularly evident among AS patients who had more severe clinical manifestations [5, 8]. The male-to-female (M : F) sex ratio in AS correlates strongly with the severity gradient of disease, which varies from 9 : 1 in the most progressive patients to a female preponderance in the mildest cases, not explained by presence of the HLA-B27 phenotype [8].

Onset of AS occurs in the first decade in less than 10 percent and after the age of 50 years in only 5% or less [6, 8]. The incidence rate rises sharply in puberty [9] or at about the adolescent age of 15 years, reaches a peak onset in the early 20s, and decreases before age 35 [8, 10]. Overall, the characteristic age-specific onset pattern of AS is little influenced by the sex effect [8, 10]. Such risk relations are consistent with the status of myofascial forces or tonicity in the body, increasing during adolescent development, peaking in early adulthood, and subsequently decreasing with aging. In the concluding section on *Avenues for future research *in an early review [6], the suggestion was raised, “also axial muscle tension may be a factor in these degenerative spinal syndromes”.

The early suspicion that axial myofascial hypertonicity could predispose to AS [6] has remained consistent with advances achieved in clinical research, immunogenetics, and molecular biology [10, 11]. However, the hypothesis has not yet been directly confirmed, due to lack of reported quantitative measurements and research on such mechanisms. Accordingly, the aims of this paper are to (1) expand upon our perspectives on the structural biomechanical concept of AS, (2) integrate mechanobiological pathways with current inflammatory concepts, (3) provide a further rationale for encouraging such research, and (4) indicate innovative techniques which may offer promise in future investigations to test the structural biomechanical hypothesis in AS [10].

## 2. Enthesis-Related Lesions as Biomechanical Links in the Ankylosing Spondylitis Process

In AS, the hallmark localization of pathological lesions is at entheses [G. *en*, in, + *thesis*, a placing; an insertion] sites, particularly in the vertebral spine [11]. Entheses are the attachments or insertions of tendons, ligaments, or joint capsules into bone [12, 13]. Enthesitis (G. *enthetos*, implanted, + -*itis*, inflammation) was defined [12] as, “traumatic disease occurring at the insertion of muscles where recurring concentration of muscle stress provokes inflammation with a strong tendency toward fibrosis and calcification.” Enthesopathy (G. *en*, in, + *thesis*, a placing, + *pathos*, suffering) was defined [12] as “a disease process occurring at the site of insertion of muscle tendons and ligaments into bones or joint capsules”. The latter term has gained favor in describing the manifold processes involving entheses, often due to chronic mechanical overloading, with or without evidence of inflammation [13].

The target enthesis lesions in AS are generally interpreted to result from inflammatory mechanisms associated with biomechanical stress at the local level [14]. The axial myofascial hypothesis proposes a centralized mechanism for increased tensional stresses in the postural musculoskeletal anatomy that can transmit excessive forces to entheses in the spine and girdles [15, 16]. A normally relaxed muscle is relatively soft and extensible. It can efficiently dissipate stress concentrations by transferring or absorbing them [13]. To the contrary, stiffer muscles less effectively absorb or distribute forces [13]. Thus, stiffer and tenser muscles could likely transmit greater stresses to tendons or ligaments and to bony enthesis sites, which serve an anchoring role [13, 15].

**Figure 1:**
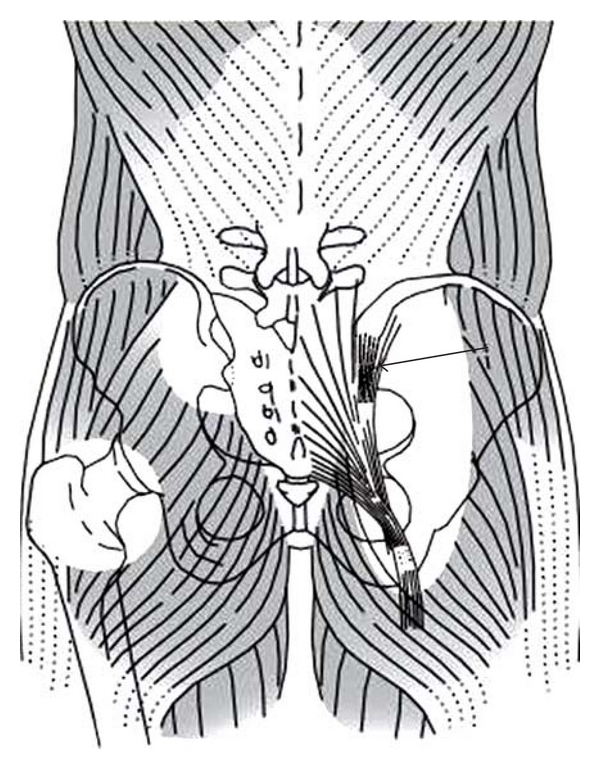
Posterior anatomical relations of the lumbopelvic region. On the left side, the upper window shows the sacroiliac joint (SIJ). The lower window shows how the hip joint is interposed between posterior cross-braced muscles and how the pelvis is stabilized. The right side shows the attachment of the hamstrings (particularly the biceps tendon) to the sacrotuberous ligament. The arrow indicates the long dorsal sacroiliac ligament (LDSIL). Axial myofascial hypertonicity could theoretically exert increased compressional forces on the pelvis, SIJs, and hips. (The original was published as Figure  14.4 in [16], In: A. Vleeming, V. Mooney, R. Stoeckart, eds. Movement, Stability, and Lumbopelvic Pain: Integration of Research and Therapy. Edinburgh, Scotland: Churchill Livingston; 2007 : 205–227, reproduced with permission from Churchill Livingston, Copyright Elsevier, 2011).

This perspective concentrates on enthesis lesions in AS, since they have received greater attention in recent research [10, 11] than a preceding review of sacroiliac joint (SIJ) involvement [16]. The mechanical principle of integrated tension (tensegrity) [17–19] is fundamental to the structural hypothesis. Application of the tensegrity theory to axial myofascial hypertonicity in the postural system can theoretically rationalize concurrently excessive tensional forces on entheses coexisting with increased compressional loads across the SIJs [16, 19]. Anatomical cross-bracing of the dorsal and lateral postural myofascial support systems could overload the SIJs by increased compressional forces and stiffness (Figure 1) [16]. Hypertonicity of the deeper vertebral myofascial system, like the lumbar multifidus muscles, could exert its main effects on increased tensional forces at the spinal entheses and manifest as symptomatic and objective postural stiffness [15].

## 3. Human Resting Muscle/Myofascial Tone (HRMT) 

Axial or postural human resting muscle/myofascial tone (HRMT) is an innate polymorphic trait which is independent of the central nervous system control and contributes vitally to stability in balanced equilibrium positions [15, 20, 21]. Increased low-back muscle stiffness and tightness in patients with early AS was first reported by Forestier et al. in 1951 [22], which was called the “bowstring sign” [22]. That observation was subsequently confirmed by palpation and electromyography studies, as reviewed [23].

The concept of HRMT is believed to incorporate an innate individual variability (polymorphism), particularly of the axial postural system [15, 24]. Variability is also likely to be clinically relevant to proximal limb muscles and girdles, but less so for the distal extremities. This paper incorporates HRMT into the biomechanical hypothesis only as related to the axial postural system effects on vertebral entheses and the SIJs [10, 16].

## 4. An Early Historical Interpretation of Clinical Syndromes at Enthesis Sites

Mechanisms causing lesions at enthesis sites are complex [13, 14]. A paucity of documentation exists on the evolution of the processes, from initiation, through their course, and into the later healing stages. The first PubMed reference found on enthesitis was from 1959, titled as “Enthesitis-traumatic disease of insertions” [25]. The tissue reactions at insertions were described as follows: “most frequently these stimuli are of traumatic, particularly microtraumatic, origin. The continually recurring concentration of muscle stress at these points provokes a reaction of inflammation with a strong tendency to the formation of fibrosis and calcification” [25].

## 5. Evolution of Histological Features in Enthesis Lesions: Stages of Erosion, Then Repair

The earliest pathoanatomical review of enthesopathy found in PubMed was from 1966 [26], which included description of both peripheral ligamentous and vertebral lesions. The enthesis was referred to as a *unit *[26] and is now referenced as an *organ* [13, 27]. The enthesis unit was defined as a special anatomical formation consisting of (1) a specifically structured part of the bone, (2) the transitional part of the tendon, and (3) interpolated fibrocartilage, which is partially calcified [26]. This definition has essentially held true [13, 27]. An absence of periosteum at the insertion sites of tendons was emphasized as well as an exceptionally great strain at the terminal part of the tendon [26]. A metaplastic process was described at the insertional zone that was considered to be a possible adaptive manifestation to “one-sided overstrain.” Often, the alterations included concurrent inflammatory changes and new bone formation, as in a reparative hyperplastic stage [26].

This group [26] described localized granular inflammatory changes in loose, richly vascularized areolar tissue of the ligamentous enthesis. At this stage, the localized bone cortex had become thinned and manifested marginal defects [26]. These findings were considered to be the destructive (*erosive*) phase of enthesitis. Notably, reparative changes were observed along with the destructive ones. In the advanced stage, typical ossifying enthesitis (enthesophyte) had developed [26].

## 6. Enthesis Lesions in Ankylosing Spondylitis

The classical reported pathoanatomical findings in AS patients are almost uniformly restricted to advanced stages in the available tissue materials, usually in late and post-mortem stages [28–32]. Initial lesions of the local processes are hardly documented [29].

The classical paper on enthesopathy in AS was the subject of the Heberden Oration, 1970 [31]. Firstly, Ball [31] compared rheumatoid spondylitis (RS), mainly defined from early cervical involved cases, to the histopathology of late AS spondylitis. In the cervical RS cases, instability and dislocation were stated to be associated with erosive synovitis of the apophyseal joints and to be associated with destructive lesions of the corresponding disc [31]. The enthesopathy of RS occurred in the annulus fibrosus of cervical discs and was considered to be secondary to the primary synovitis. It was not associated with prominent reactive bone formation, as was found in AS [31]. Immobilization was considered as a mechanism in the prominent enchondral ossification feature in AS, which occurs in the apophyseal joints [31]. 

Secondly, Ball [31] examined extraspinal ligamentous lesions of AS in mainly biopsy specimens. Like Niepel et al. [26], the striking findings were multiple focal microscopic inflammatory lesions localized to the ligamentous attachments [31]. The whole or greater part of the enthesis was destroyed in association with small erosions or defects in the cortical bone. The inflammation reaction was considered brief in individual lesions, mostly involving lymphocytes and plasma cells [31]. The erosive lesions healed by deposition of reactive (woven) bone in a finely fibrous connective tissue without preceding cartilage formation. The new bone tended to fill in the cortical defect as well as having joined the deeper bone to the eroded end of the ligament. The new bone formed a new enthesis above the original level of the cortical surface. In the final healing stage, it appeared as a small irregular bony prominence [31].

## 7. Intervertebral Disc Lesions in Ankylosing Spondylitis

According to Ball [31], the concept that ossification of the intervertebral discs in AS is inflammatory in origin may be traced back to Engfeldt et al. [28] and to Bywaters [29, 30]. Currently, inflammation is still considered to be essential to the pathogenesis of AS [10, 11]. Ball [31] interpreted the spinal lesions from 13 necropsy specimens, 12 having deceased after 12 to 33 years following onset of AS. The late and somewhat fragmentary nature of the spinal specimens was acknowledged [31].

Ball [31] found erosive lesions at the anterior or antero-lateral attachments of the outer fibers of the annulus at the corner of the vertebral body and at the junction of the annular flange. Infiltration of lymphocytes and plasma cells was scanty in some and prominent in other lesions. Of note, the common longitudinal ligament was conspicuously unaffected in anterior lesions. In the area of the erosive lesions, the vertebral cortex was replaced by a narrow layer of reactive bone that spread for a short distance into the outer annulus, that is, the disc syndesmophyte. The process involved the replacement and remodeling of the reactive bone into mature (lamellar) bone. Ball [31] tentatively interpreted a mechanism for the growth of disc syndesmophytes as “the intermittent occurrence of inflammatory lesions in previously stable syndesmophytes.” Our tentative biomechanical interpretation of this process might be that an inflammatory phase, possibly contributed by microinjury, precedes the ossification (healing) or syndesmophyte (immobilization) stage.

Lastly, Ball [31] considered the ossification process in the apophyseal joints. In two AS cases, capsular ossification occurred in the setting of doubtful erosive synovitis and with preserved articular cartilages. The capsular findings were interpreted as equivalent to syndesmophyte formation and that the enclosed articular cartilages were being replaced by a nonspecific process of enchondral ossification [31].

In subsequent reviews of the articular pathology of ankylosing spondylitis, Ball [32–34] raised the possibility that inflammatory enthesopathy was not the only mechanism that may give rise to syndesmophyte formation. This process can be attributed to implied immobilization and changes in load transmission across the disc [33]. Destructive spondylodiscitis lesions were also reviewed in the later articles [32, 33], which had not been earlier considered [31]. Clinical, radiological, and pathological evidence indicated that these destructive spondylodiscitis lesions are essentially owing to trauma in a spine that for various reasons is susceptible to stress [32, 33]. Controversy exists, however, regarding an interpretation of the variable histopathology of the spondylodiscitis lesions, which may not show inflammatory infiltration [32, 33].

## 8. A Novel Biomechanical Interpretation of Classical Histological Studies of Enthesis Lesions

Injury mechanisms from structural impacts were only later considered by Ball [32, 33]. Microinjury was not addressed in relation to causing erosive lesions at the attachment sites of the annulus fibrosus to the vertebral margins, at which syndesmophytes start to grow [31, 32]. Modern biomechanical research has established that this disc-vertebral interface is subjected to increased localized force concentrations at its outer circumferential boundaries in the spine [35].

Further research into the indicated differential involvement of the lumbar anterior longitudinal ligament (ALL) versus the disc entheses in AS [28, 31] could potentially clarify mechanistic pathways in syndesmophyte formation. Many reports stated that the anterior ossification between the vertebral bodies is not situated in the ALL, but rather in the periphery of the disks [28, 31]. Both tissues are in close proximity to the cellular infiltrations and new bone formation at the edge of the annulus [31, plate 3(a)]. A hypothetical question might be raised as follows. Could differential degrees of mechanically induced microinjury, in one versus the other tissue, lead to the observed differences in inflammatory reactions? If so, might such mechanisms influence later bony proliferation leading to differential involvements in syndesmophyte formation? Such biomechanical issues had not been raised in the classical papers [28–33] nor have we encountered them in the current literature.

## 9. Parallels in Tendinopathy and Ankylosing Spondylitis Enthesis Lesions

Tendinopathy is currently considered to be mainly noninflammatory (enthesopathy) [13, 36, 37], whereas AS is thought to have a primarily inflammatory cause (enthesitis) [11]. Mechanical overloading is believed to be the primary initiating pathology in degenerative and overuse tendinopathy [38, 39]. Like AS [40], many tendinopathies are insertional in nature, having a disease localization at enthesis sites, where the tendon or ligament attaches to bone [37]. One may question if the pathways leading to these conditions might be more similar than previously believed. Might biomechanical stress mechanisms also be an important predisposing pathway in AS?

Histopathological studies of the Achilles tendinopathy are typically characterized by a lack of inflammatory cells and other evidence for inflammatory mechanisms [13, 41, 42]. Rather, they show a poor healing response, involving collagen fiber disorientation, tenocyte hypercellularity, neovascularization, and neurovascular ingrowth [38, 42]. Calcification and osseous metaplasia can also be found in symptomatic tendons [38]. A primarily noninflammatory mechanism is also endorsed by the limited efficacy of anti-inflammatory drug therapy of tendinopathy [43].

Bone formation can occur at enthesis (enthesophyte) sites in tendinopathy disorders and differs from normal fracture healing [41]. Enthesophytes have been shown to form in regions of high tensile forces, thereby increasing contact surface area between the tendon or ligament and bone [44, 45]. Anatomical connections from entheses to neighboring synovium and bone may explain how chronic biomechanical stress at these sites may lead to secondary histopathology in AS, including synovitis and bone marrow edema [44–46].

## 10. Clinical and Cytokine Parallels in Tendinopathy and Ankylosing Spondylitis

Mechanical forces interacting with cytokines, such as TGF-*β* and IL-1*β*, can stimulate extracellular matrix (ECM) gene and protein expression, leading to either anabolic or catabolic pathways [47]. Excessive mechanical forces may result in tissue microinjury in tendinopathy and in AS. Such stress-mediated processes could activate inflammatory responses by release of damage-associated molecules, cytokines, or other mediators [44, 48]. In overuse tendinopathy, an early increase in proinflammatory cytokine levels is believed to contribute to the observed later-stage degenerative changes [49]. Few human data are available on cytokine influences upon enthesis lesions in AS.

Athletes with symptomatic tendinopathy have been shown to have stiffer Achilles tendons than normal control subjects [50]. Individuals with diabetes mellitus are also predisposed to the Achilles tendinopathy, possibly related to structural abnormalities associated with increased stiffness [51]. Perhaps, greater myofascial stiffness could generally predispose individuals to enthesopathy, including tendinopathy and AS [13, 15, 37]?

## 11. Comparison of Demographic Profiles and Implications of Treatment Responses

Similarities in host profiles of TP and AS patients could lend support to biomechanical pathways in both disorders. Overall, AS and patellar tendinopathy occur about twice as often in men as in women [8, 38, 39, 52]. Men have greater musculotendinous stiffness than women, which correlated positively with muscle mass [53]. Men have greater predisposition to bony tensional attachment injuries, whereas women are more susceptible to joint instability and intra-articular injury, such as anterior cruciate ligament (ACL) rupture [53, 54].

Although inflammation is not considered to be the primary cause of tendinopathy, local morning stiffness which eases with activity is commonly observed [38]. Such symptoms are analogous to the pattern of chronic *inflammatory-type *back pain in AS [55]. Spinal mobility is restricted in early AS [56] and reduced muscular flexibility is also thought to contribute to the development of patellar tendinopathy in athletic populations [57].

Recommendations for management of tendinopathy [13, 43] and AS [58–60] both include physical therapy interventions to maximize the long-term quality of life, although anti-inflammation therapy has an added, relatively greater benefit in AS than in tendinopathy [11, 58]. The combination of regular, active exercise and pharmacological treatment has been shown to be more effective in the long-term than usual therapy in AS [59]. Thus, physical rehabilitation approaches are beneficial in both conditions. The difference in benefits from anti-inflammatory therapy between TP and AS may reflect relative degrees of biomechanical versus immunological pathways in the respective conditions [10, 11].

If increased axial myofascial tonicity is confirmed in AS [10, 22, 23], then the transmitted enthesis tissue stiffness could result in greater microinjury. Such pathway deserves further investigation and is consistent with beneficial results of structured rehabilitation interventions in AS, such as global postural reeducation [61] and active exercise treatments [59].

## 12. Mechanobiology and Immunobiology Interactions in Ankylosing Spondylitis: Figure 2

Trauma was proposed as a possible initiating stimulus for chronic interactions of the innate and adaptive arms of the immune system in spondyloarthritis (SpA) and AS [14, 48, 62]. Tissue microinjury could locally activate the innate immune system by release of damage-associated molecules, cytokines, or other mediators [44, 45, 62]. In normal subjects, proinflammatory cytokine release is increased in skeletal muscle following eccentric exercise [63]. However, in the same normal subjects, a circulating systemic proinflammatory response is not seen, possibly due to activation of anti-inflammatory cytokines [63].

**Figure 2:**
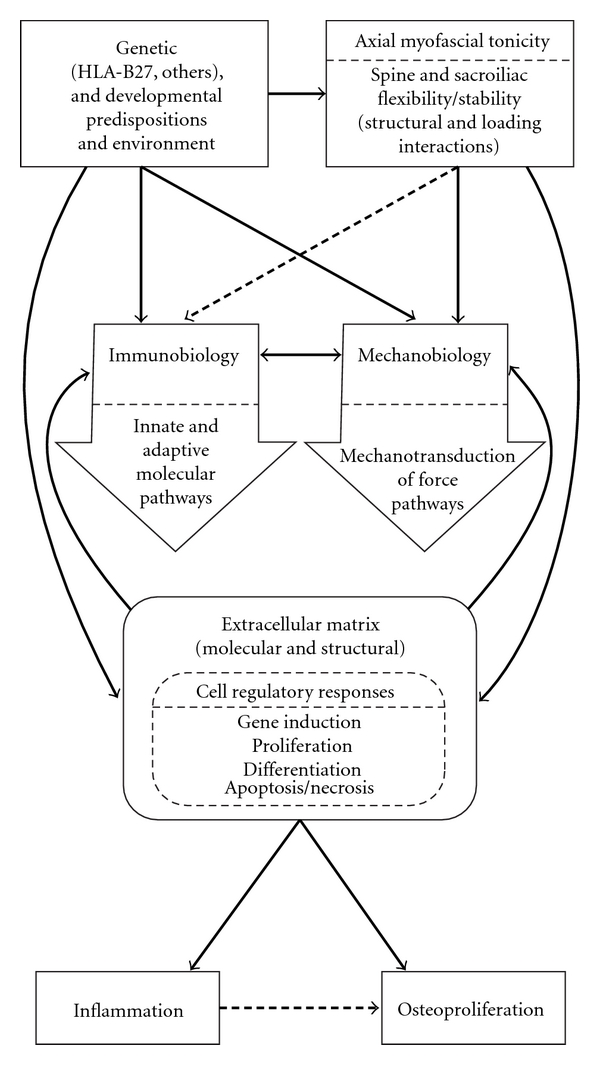
A theoretical outline of immunobiology and mechanobiology processes interacting in initiation and perpetuation of pathways in ankylosing spondylitis. (Reproduced with permission for reprinting from the Journal of Rheumatology; the original was published as a Figure in [10], A. T. Masi, 2011; 38 (10): (pp. 2092–2094)).

We agree with others [14, 48, 64] that microtrauma and biomechanical stress may be important initiating triggers and chronic perpetuating stimuli in AS. Our hypothesis of an innate axial (spinal) myofascial hypertonicity [10, 15, 16] provides a theoretical framework for increased axial stiffness in AS. In turn, it could predispose to exaggerated stress transmissions through entheses, leading to greater micro-damage and abnormal tissue repair responses [15, 16, 47]. A constitutional biomechanical diathesis, in combination with a proinflammatory predisposition and other genetic factors, could initiate and perpetuate inflammation as well as osteoproliferation (syndesmophyte) formation in the AS patient (Figure 2) [16, 64, 65].

Mechanotransduction translates intrinsic and extrinsic forces into cellular and molecular responses [18, 47, 66]. Accordingly, cells modify their responses to varied stress by mechanobiology pathways [66]. Increased structural and entheseal stresses on extracellular matrix (ECM), tendon, and bone [16, 66] of AS patients could be expected to result in altered and pathological tissue responses [15, 16, 37, 44, 66]. In an *in vivo* rodent tendon model, mechanical stress can alter the expression of IL-1*β* in a load-dependent fashion [67]. In bone, osteoblasts are also load-sensitive cells and can produce bone morphogenic proteins (BMPs) [66], which were found to be increased in a murine model of ankylosing enthesitis [64, 68]. Moreover, microtrauma can release cartilaginous molecules that activate inflammation by pattern recognition receptors [48, 69]. Those released ECM fragments can be incorporated intracellularly and initiate a proinflammatory cascade [69].

## 13. Complex Interactions of Wnt, DKK-1, BMPs, and TNF in Murine Models

Investigation into TNF mechanisms in the complex bony proliferation process has revealed relations to other molecular pathways. The Wnt family members are relevant, as some induce differentiation of osteoblasts and block osteoclast activity, resulting in a net gain of bone [70]. Dickkopf-1 (DKK-1) is an antagonist of the Wnt/*β*-catenin bone-forming pathway. Excess TNF, as commonly occurring in AS patients, has been found to stimulate the production of DKK-1 [71]. In a murine inflammatory arthritis model, DKK-1 inhibition reversed bone erosion into osteophyte formation, suggesting that DKK-1 is an important inhibitory molecule in bone formation [71]. Another study found *increased* serum DKK-1 levels in AS patients treated with anti-TNF agents as compared to rheumatoid arthritis patients and normals [72]. These findings indicate a paradox in the osteoproliferation mechanisms in AS.

The TNF-brake hypothesis [73] may offer a possible explanation of the bony proliferative process in AS. It proposes that, after TNF blocking agents are administered, the DKK-1 is less stimulated by TNF, and then Wnt family members are less inhibited, permitting increased bony proliferation [73]. It has been further proposed that, with increase of Wnt member signaling, DKK-1 may be increased as a *feedback* balancing response [72].

Bone morphogenetic proteins (BMPs) also have a role in osteoproliferation. The BMPs control bone growth and are inhibited by noggin gene transfer [68]. TNF can also stimulate certain BMPs [68]. Accordingly, overexpression of BMPs may stimulate greater bone formation at damage sites and may contribute to osteophyte formation [74]. Since AS patients may have increased TNF levels, BMP could be upregulated and may contribute to bony proliferation. One study found a nonsignificant increase in serum BMP-7 levels in AS patients [75], and increased serum levels of BMP-2 and BMP-7 were found in another study of AS patients [76].

## 14. Mechanical Stress Activation of Inflammatory Pathways

Mechanical stress can become translated to inflammation and cellular responses via molecular induction pathways [18, 47, 66]. Cyclooxygenase-2 (Cox-2) can be induced by mechanical stress, which leads to increased prostaglandin E2 (PGE2) synthesis in cartilage, as commonly seen in OA [77]. Also, the intracellular NALP3 pathway can translate mechanical or metabolic stimuli into cellular responses [69]. In this pathway, mechanical or metabolic stress can release molecules related to cellular damage. These cellular stress molecules can be sensed by membrane receptors or be endocytosed, leading to NALP3 activation. This molecule is part of the nucleotide-binding domain, leucine-rich repeat containing family [69]. It is related to increases in proinflammatory cytokines, such as TNF [69]. This pathway culminates in increased levels of the proinflammatory cytokine, interleukin-1*β* [69].

These Cox-2 and NALP3 transduction pathways may possibly explain how increased mechanical loading may lead to inflammation and bony proliferation. Both pathways induce increased proinflammatory cytokine release [69, 77] and are consistent with increased BMP upregulation. In a review, Lories et al. [64] proposed that TNF stimulates BMP and DKK-1. While DKK-1 inhibits osteoblast differentiation by suppressing Wnt family members, the BMP augmentation may be an even greater stimulus for bone formation [64]. One study indicated that anti-TNF treatment was more clinically effective in patients with shorter disease duration [78]. It was suggested that disease modification is required at the earliest stages possible to avoid increased levels of BMP or sensitization to Wnt members and greater osteoproliferation [78]. 

Recent research has indicated that tissue inflammatory reactions, as is found in rheumatoid arthritis (RA) and in AS lesions, can result from nonspecific cytokine stimulation, including T-helper cell activation [79]. Accordingly, such tissue reactions need not to be autoimmune nor dependent upon specific antigen activations. Rather, such inflammatory reactions in these diseases may be initiated and contributed by cytokines, possibly released from micro injury contributed by an innate biomechanical diathesis. In such scenario, excessive axial HRMT could be a structural body variant that may predispose to enhanced entheseal microinjury and initiation of inflammatory pathways [10, 15]. Of potential interest, a recent report indicated marked (up to 100-fold) upregulation of gene transcripts related to myocyte/myofibroblast biology in synovial tissue samples from SpA versus RA arthritis patients, which suggested structural remodeling mechanisms in SpA [80].

## 15. Significance of Research on Axial HRMT and Critical Barriers to Progress

Myofascial tone is literally the *tension* or *stiffness* of these specialized tissues which is universally recognized to accompany movements and resistive activities. In the axial (spinal) system, myofascial tone is the primary contributor to stability in various postural activities, far greater than the osteoligamentous component [15, 20]. Myofascial tone exists in the body, whether it is (1) passive or active (2) in postural balance or unbalanced, and (3) static (resting) or in motion. Accordingly, the body may be considered as being in a pre-stressed architectural design, which is labeled as biotensegrity [17, 18]. Notably, myofascial tone may encompass intrinsic passive properties of the tissues as well as an active contractile component which is superimposed under the control of the central nervous system (CNS) [15, 19–21]. The passive, resting (static) tone is independent of CNS control. It results from the elastic mechanical properties of the stable cross-bridges between the actin and myosin filaments of muscle fibers and the integrated connective tissue filaments [15, 20, 21].

A critical problem in the interpretation of clinical studies of HRMT has been the differentiation of the static, passive condition alone from any additional low-level contraction superimposed by CNS activation [81]. Such confounding of the passive properties by the low-level active contraction component has led to confusion and misinterpretation in studies of the human physiology and clinical relevance of myofascial tone [15, 20, 21]. We endorse research to accurately characterize the low-level, passive biomechanical properties of axial HRMT (EMG-silent) in relation to AS risks. The intrinsic HRMT viscoelastic property is a vital body trait which has been overlooked [82] or misinterpreted [15, 20, 21]. Current lack of confirmed quantitative data on individual variability (polymorphism) of axial HRMT now warrants critical study of its physical properties and relevance to body biomechanics in AS.

## 16. Quantifying Axial HRMT (EMG-Silent)

Emerging methodological techniques can noninvasively quantify the viscoelastic (elasticity, stiffness, and tension) properties of myofascia at precise body levels. Such measurements have not yet been reported at the lumbar level. Concurrent surface electromyography (sEMG) should be performed at the measurement loci to confirm the passive status and absence of superimposed CNS activation. Extensive data exist on spinal muscle properties in various degrees of activated contraction, but none for comparison at rest [82].

The lumbar spine is stabilized and moved by sets of muscles that have varying biomechanical roles [15, 83]. The core muscles provide mainly segmental stability, whereas more peripheral muscles control mainly global movements and active stabilization of the trunk [15, 83, 84]. Based upon such biomechanical principles, our hypothesis is that the core muscles, for example, lumbar multifidus, will contribute mainly to variability in innate spinal myofascial stiffness among individuals (polymorphism). Some polymorphic variation is also expected in the more superficial muscle networks, for example, erector spinae.

Techniques have existed that can quantify muscle tone [81, 85]. However, they are basically dynamic, operating at macroanatomical levels via imposed passive joint movements (calf, thigh, or the trunk). Emerging technology, like the Myoton instrument [86, 87] and ultrasonic shear wave elastography [50, 88–90] will enable noninvasive quantification of resting muscle properties of elasticity, stiffness, and tension at precise lumbar anatomical levels. The Myoton range of depth measurements is mainly limited to several centimeters within the more superficial muscles, for example, erector spinae. Ultrasonic shear wave elastography technique will be required to measure the deeper or core lumbar muscles, for example, the multifidus [90]. The use of separate measurement techniques on the superficial (Myoton and elastography) and deeper (elastography) lumbar muscles will aid in interpreting the respective findings.

Cross-sectional analysis of axial HRMT could test whether or not AS patients have greater lumbar myofascial biomechanical properties of stiffness/tension than normal subjects or even greater than patients with non-AS chronic, mechanical low-back disorders [15]. The ultrasonic shear wave elastography technique is able to scan deeply into the dorsolumbar myofascial tissues to investigate viscoelastic properties of the multifidus at the L3-L4 level and of its enthesis sites. These viscoelastic properties have not been reported and may become valuable as a biomarker in an earlier diagnosis of AS. Cost-effective biomechanical criteria for AS have not yet been investigated, which may help to identify its early stage [22, 23]. Viscoelastic properties may have utility in also helping to identify asymptomatic high-risk susceptibles who may be likely to develop AS, such as those HLA-B27-positive first-degree relatives of AS probands [15].

## 17. Technical Innovation Is Needed to Quantify Biomechanical Influences on AS

Interdisciplinary biomechanical approaches are needed to quantify and analyze passive (EMG-silent) axial (spinal) myofascial tone in normal subjects, patients with AS, and those with other chronic LBP conditions. Quantitative biomechanical study is needed to analyze individual variability characteristics (polymorphisms) of the hardly appreciated axial HRMT trait in large samples of healthy persons. Such studies will reveal and elucidate the significance of resting axial muscle tone in the spinal musculoskeletal system.

When axial (and general body) passive tone and stiffness are normally sufficient, this trait is biomechanically and metabolically efficient in energy expenditure [19]. Insufficient or excessive axial HRMTs are likely to have physical penalties, like increased risks of developing spinal consequences [24] and possible energy metabolic impacts [19]. Axial HRMT is a macrostructural component of the body tensegrity design [17–19]. Incorporation of tensegrity [17–19] concepts and utilization of structural mechanical modeling [35] promises to enhance understanding of spinal disorders, like AS.
